# Dynamic regulation of fatty acid pools for improved production of fatty alcohols in *Saccharomyces cerevisiae*

**DOI:** 10.1186/s12934-017-0663-3

**Published:** 2017-03-15

**Authors:** Paulo Gonçalves Teixeira, Raphael Ferreira, Yongjin J. Zhou, Verena Siewers, Jens Nielsen

**Affiliations:** 1 Department of Biology and Biological Engineering, Chalmers University of Technology, 412 96 Gothenburg, Sweden; 2 Novo Nordisk Foundation Center for Biosustainability, Chalmers University of Technology, 412 96 Gothenburg, Sweden; 3 Novo Nordisk Foundation Center for Biosustainability, Technical University of Denmark, 2800 Kgs. Lyngby, Denmark

**Keywords:** Fatty alcohols, Fatty acid activation, *FAA1*, Dynamic control, Yeast, Metabolic engineering

## Abstract

**Background:**

In vivo production of fatty acid-derived chemicals in *Saccharomyces cerevisiae* requires strategies to increase the intracellular supply of either acyl-CoA or free fatty acids (FFAs), since their cytosolic concentrations are quite low in a natural state for this organism. Deletion of the fatty acyl-CoA synthetase genes *FAA1* and *FAA4* is an effective and straightforward way to disable re-activation of fatty acids and drastically increase FFA levels. However, this strategy causes FFA over-accumulation and consequential release to the extracellular medium, which results in a significant loss of precursors that compromises the process yield. In the present study, we aimed for dynamic expression of the fatty acyl-CoA synthetase gene *FAA1* to regulate FFA and acyl-CoA pools in order to improve fatty alcohol production yields.

**Results:**

We analyzed the metabolite dynamics of a *faa1Δ faa4Δ* strain constitutively expressing a carboxylic acid reductase from *Mycobacterium marinum* (MmCAR) and an endogenous alcohol dehydrogenase (Adh5) for in vivo production of fatty alcohols from FFAs. We observed production of fatty acids and fatty alcohols with different rates leading to high levels of FFAs not being converted to the final product. To address the issue, we expressed the MmCAR + Adh5 pathway together with a fatty acyl-CoA reductase from *Marinobacter aquaeolei* to enable fatty alcohol production simultaneously from FFA and acyl-CoA, respectively. Then, we expressed *FAA1* under the control of different promoters in order to balance FFA and acyl-CoA interconversion rates and to achieve optimal levels for conversion to fatty alcohols. Expressing *FAA1* under control of the *HXT1* promoter led to an increased accumulation of fatty alcohols per OD_600_ up to 41% while FFA levels were decreased by 63% compared with the control strain.

**Conclusions:**

Fine-tuning and dynamic regulation of key metabolic steps can be used to improve cell factories when the rates of downstream reactions are limiting. This avoids loss of precursors to the extracellular medium or to competing reactions, hereby potentially improving the process yield. The study also provides knowledge of a key point of fatty acid regulation and homeostasis, which can be used for future design of cells factories for fatty acid-derived chemicals.

**Electronic supplementary material:**

The online version of this article (doi:10.1186/s12934-017-0663-3) contains supplementary material, which is available to authorized users.

## Background

Society’s need for sustainable production of liquid fuels and oleochemicals is indisputable. The use of plants for the extraction of lipid molecules for conversion to biofuels and oleochemicals is often not sustainable in a long term due to requirements for large areas of fertile land together with extensive extraction and chemical conversion processes. Therefore, there is a need for alternative production routes for petrol and plant lipid-derived chemicals that can simultaneously offer a sustainable life cycle and assure a stable supply. The development of microbial cell factories proposes a substitute production path by redirecting cell metabolism towards a product of interest [1, 2]. Many studies have already shown how *Saccharomyces cerevisiae* can be successfully engineered for production of different fatty acid-derived chemicals such as fatty alcohols, alkanes, alkenes, fatty acyl ethyl esters and triacylglycerols [3–6].

For an efficient production of a desired chemical through fermentation, there is usually a need to engineer the production organism for reduced formation of byproducts. Byproduct formation diverts nutrients towards unwanted molecules, reducing the yield of the desired product and compromising the process efficiency [7–9]. In metabolic engineering, byproduct formation easily arises when fluxes are not properly balanced and pathway precursors or intermediates accumulate in the cell, becoming abundant substrates for side reactions and processes. For this reason, balancing fluxes through enzyme modulation and controlling metabolite pool levels usually play an important role with regard to the final product yield.

Fatty alcohols in *S. cerevisiae* can be produced either from acyl-CoA or free fatty acids (FFAs) in two steps. In either case, the precursor is first converted to a fatty aldehyde through an acyl-CoA reductase [10] for acyl-CoA reduction, or a carboxylic acid reductase [11] for FFA reduction. The formed fatty aldehydes are then reduced to a primary alcohol by endogenous alcohol dehydrogenases [12]. An alternative route for fatty alcohol production from acyl-CoA is a four-electron reduction catalyzed by a bifunctional fatty acyl-CoA reductase [13], in which an aldehyde is also formed as an intermediary metabolite but the enzyme is capable of catalyzing both reaction steps.

The most successful strategies for production of fatty alcohols so far rely on using the FFA pathway, in which the main factor of success is the possibility to accumulate FFAs in the cytosol at levels several orders of magnitude higher compared with acyl-CoA [14–16]. Here, one of the major strategies used for accumulation of FFAs is the simultaneous deletion of the fatty acyl-CoA synthetase genes *FAA1* and *FAA4*, encoding the main responsible enzymes for the activation of FFAs to fatty acyl-CoA [17, 18]. Simultaneous deletion of these two genes together with *POX1* (encoding fatty acyl-CoA oxidase, the first enzyme of the fatty acid beta-oxidation pathway), allows production of up to 490 mg/L of FFA, whereas the wild-type reference strain only produces around 3 mg/L FFAs [5, 19]. Although there remains some discussion as to the origin of these fatty acids, evidence shows that these are produced mainly from hydrolysis of acyl chains from storage and membrane lipids, and not directly from acyl-CoA due to thioesterase activity or spontaneous hydrolysis [18].

Increased FFA production in *faa1Δ faa4Δ* deletion strains results in high levels of these FFA accumulating in the extracellular medium. Further increasing production levels in these same strains results in an increase of extracellular FFA while intracellular levels are not significantly changed, which strongly suggests a limit to intracellular FFA accumulation in the cell [5, 19]. It is unclear if this release of FFA to the medium is carried out by uncharacterized transporters or if it is a process of transmembrane diffusion due to very high concentration of FFAs in the cytosol. In either case, released FFA are inaccessible to the cell due to lack of re-import and fatty acid activation mechanisms, for which *FAA1* and *FAA4* are responsible [18, 20, 21].

Although an efficient accumulation of FFAs is most beneficial for the production of chemicals derived thereof, enzymes identified so far for production of fatty acid-derived chemicals such as fatty alcohols are often not efficient enough to keep up with the flux of FFA formation. This creates a situation where FFAs are produced faster than they can be converted to the final product [5].

In order to qualify as an industrially feasible process, production of fatty acid-derived chemicals requires achieving the maximum production yield possible. Thus far, these unbalanced fluxes create an intra- and extracellular over-accumulation of a large amounts of FFAs, which are ultimately not converted to the product of interest. This constitutes a serious loss of carbon and reducing cofactors that might hinder the production yields for fatty alcohols and other fatty acid-derived chemicals.

We therefore studied how fine-tuning of a single step in this pathway would allow control of the involved metabolite levels and how this could be a possible approach to reduce the described problem. A successful application of this strategy would allow for an improvement in final product yields and production titers since it provides an optimization of resource usage during cultivation of engineered cell factories.

## Methods

### Plasmid construction

The *FAA1* gene was amplified by PCR from *Saccharomyces cerevisiae* CEN.PK113-11C and cloned into p413TEF using restriction enzymes BamHI and XhoI, resulting in plasmid pTEF-*FAA1*. For construction of plasmids pHXT1-*FAA1* and pHXT7-*FAA1*, the *HXT7* and the *HXT1* promoters were amplified from *S. cerevisiae* CEN.PK113-11C and fused to the backbone p413 by PCR. The p413 backbone plasmid was amplified from p413TEF. The *FAA1* gene and *HXT1/HXT7* promoters were amplified by PCR to generate the complementary overhangs for insertion into the plasmid by Gibson assembly (New England Biolabs, Ipswich, Massachusetts, United States). Strains and plasmids generated and used in this study are presented in Table 1 and Table 2, respectively. A list of primers used for plasmid construction is shown in Additional file 1: Table S1.

**Table 1:** *Saccharomyces cerevisiae* strains used in this study

Strain	Genotype	References
YJZ08	MATa MAL2‐8c SUC2 *his3Δ1 ura3‐52 hfd1Δ pox1Δ faa1Δ faa4Δ*	Zhou et al. [5, 6]
YZFOH1	YJZ08 pAOH3	This study
YZFOH2	YJZ08 pAOH9	This study
p413	YJZ08 pAOH9 p413	This study
*HXT1p*-*FAA1*	YJZ08 pAOH9 pHXT1-FAA1	This study
*HXT7p*-*FAA1*	YJZ08 pAOH9 pHXT7-FAA1	This study
*TEF1p*-*FAA1*	YJZ08 pAOH9 pTEF1-FAA1	This study
*CUP1p*-*FAA1*	YJZ08 pAOH9 pCUP1-FAA1	This study

**Table 2:** Plasmids used in this study

Plasmid	Genotype/features	References
pYX212	pYX212 empty plasmid: 2 μm, ampR, URA3, TPIp, pYX212t	R&D systems
pAOH3	pYX212-(TPIp-*npgA*-FBA1t)-(TDH3p-*MmCAR*-ADH1t)-(TEF1p-*ADH5*-pYX212t)	Zhou et al. [5, 6]
pAOH9	pYX212‐(TPIp‐*npgA*‐FBA1t)-(TDH3p‐*MmCAR*‐ADH1t)-(tHXT7p‐*ADH5*-CYC1t)-(TEF1p‐*FaCoAR*‐pYX212t)	Zhou et al. [5, 6]
p413	p413TEF empty plasmid: CEN.ARS, ampR, HIS3, TEF1p, CYC1t	ATCC^®^ 87362
pTEF1-FAA1	p413-TEF1p-*FAA1*-CYC1t	This study
pHXT1-FAA1	p413-HXT1p-*FAA1*-CYC1t	This study
pHXT7-FAA1	p413-HXT7p-*FAA1*-CYC1t	This study
pCUP1-FAA1	p413-CUP1p-*FAA1*-CYC1t	This study

### Growth medium

*Saccharomyces cerevisiae* strains with uracil and histidine auxotrophies were grown on YPD plates containing 20 g/L glucose, 10 g/L yeast extract, 20 g/L peptone from casein and 20 g/L agar. Plasmid carrying strains were grown on selective growth medium containing 6.9 g/L yeast nitrogen base w/o amino acids (Formedium, Hunstanton, UK), 0.77 g/L complete supplement mixture w/o histidine and uracil (Formedium), 20 g/L glucose and 20 g/L agar. Shake flask cultivations were performed in minimal medium containing 20 g/L glucose, 5 g/L (NH_4_)_2_SO_4_, 14.4 g/L KH_2_PO_4_, 0.5 g/L MgSO_4_·7H_2_O. After sterilization, 2 mL/L trace element solution and 1 mL/L of vitamin solution were added. The composition of the trace element and vitamin solution has been reported earlier [32].

### Shake flask cultivations

All experiments were performed with strains cultivated as biological triplicates. This means that three independent transformants were used to start pre-cultures. For these, 3 mL of minimal medium in a 15 mL tube, or in 5 mL in a 50 mL tube, were inoculated for the first experiment, and cultivated at 200 rpm and 30 °C for 18 h. Subsequently, the pre-culture was used to inoculate 20 mL of minimal medium in a 100 mL shake flask, or 100 mL of minimal medium in a 500 mL shake flask for the first experiment, at an OD_600_ of 0.1. Shake flasks were incubated at 200 rpm and 30 °C for 72 h.

A spectrophotometer (Genesis20, Thermo Fisher Scientific, Waltham, MA, USA) was used to measure cell growth at designated time points and at the end of the shake flask cultivations. Optical density (OD_600_) was measured by absorbance at 600 nm of a diluted culture sample.

Growth rates on glucose were calculated using OD_600_ data points between 6 and 12 h, from which the slope of the plotted log (OD_600_) values over time interval was calculated.

### Quantification of extracellular metabolites

For quantification of glucose and ethanol, 1 mL samples were taken throughout the culture. The biomass was removed by filtration using a 0.45 µm nylon filter (VWR International AB, Stockholm, Sweden). Sample analysis was performed by HPLC using a Dionex Ultimate 3000 (Dionex, Sunnyvale, CA, USA) together with an Aminex HPX-87H column (300 × 7.8 mm, Bio-Rad Laboratories, Hercules, CA, USA) and a refractive index detector (512 µRIU). The column temperature was kept constant at 45 °C and 15 µL were injected into the mobile phase consisting of 5 mM H_2_SO_4_. The flow rate was set to 0.6 mL/min.

### Quantification of lipids

Samples for lipid analysis were taken as 5 mL of culture at the end of the shake flask cultivations, after 72 h. Subsequently, the samples were centrifuged at 4000 rpm and the supernatant was discarded. The pellets were kept at −20 °C for 5 min and then freeze-dried using a Christ alpha 2–4 LSC (Christ Gefriertrocknungsanlagen, Osterode, Germany). The samples were analyzed as described previously [33] using 10 mg of dry cell biomass.

### Quantification of FFAs and fatty-alcohols

FFAs were simultaneously extracted and methylated by dichloromethane containing methyl iodide as methyl donor [34]. Briefly, 200 μL aliquots of whole cell culture (cells + supernatant) were taken into glass vials, then 10 μL 40% tetrabutylammonium hydroxide (base catalyst) was added immediately followed by addition of 200 μL dichloromethane containing 200 mM methyl iodide as methyl donor and 100 mg/L pentadecanoic acid as an internal standard. The mixtures were shaken for 30 min at 1400 rpm. By using a vortex mixer, and then centrifuged at 5000*g* to promote phase separation. A 160 μL dichloromethane layer was transferred into a GC vial with glass insert, and evaporated 4 h to dryness. The extracted methyl esters were resuspended in 160 μL hexane and then analyzed by gas chromatography (Focus GC, ThermoFisher Scientific) equipped with a Zebron ZB-5MS GUARDIAN capillary column (30 m × 0.25 mm × 0.25 μm, Phenomenex) and a Flame Ionization Detector (FID, ThermoFisher Scientific). The GC program was as follows: initial temperature of 50 °C, hold for 2 min; ramp to 140 °C at a rate of 30 °C per minute, then raised to 280 °C at a rate of 10 °C per min and hold for 3 min. The temperature of inlet was kept at 280 °C. The injection volume was 1 μL. The flow rate of the carrier gas (helium) was set to 1.0 mL/min. Final quantification was performed using the Xcalibur software.

For fatty alcohol quantification, cell pellets were collected from 5 mL cell culture and then freeze-dried for 48 h. Metabolites were extracted by 2:1 chloroform:methanol solution [33], which contained pentadecanol as internal standard. The extracted fraction was dried by rotary evaporation and dissolved in ethyl acetate. Quantification of fatty alcohols was performed on the same GC–FID system as used for fatty acid analysis. The GC program for fatty alcohol quantification was as follow: initial temperature of 45 °C hold for 2 min; then ramp to 220 °C at a rate of 20 °C per min and hold for 2 min; ramp to 300 °C at a rate of 20 °C per min and hold for 5 min. The temperature of the inlet was kept at 250 °C. The injection volume was 1 μL. The flow rate of the carrier gas (helium) was set to 1.0 mL/min. Final quantification was performed with Xcalibur software.

## Results

### FFA accumulation during fatty alcohol production in a *faa1Δ faa4Δ* strain

**Fig. 1:**
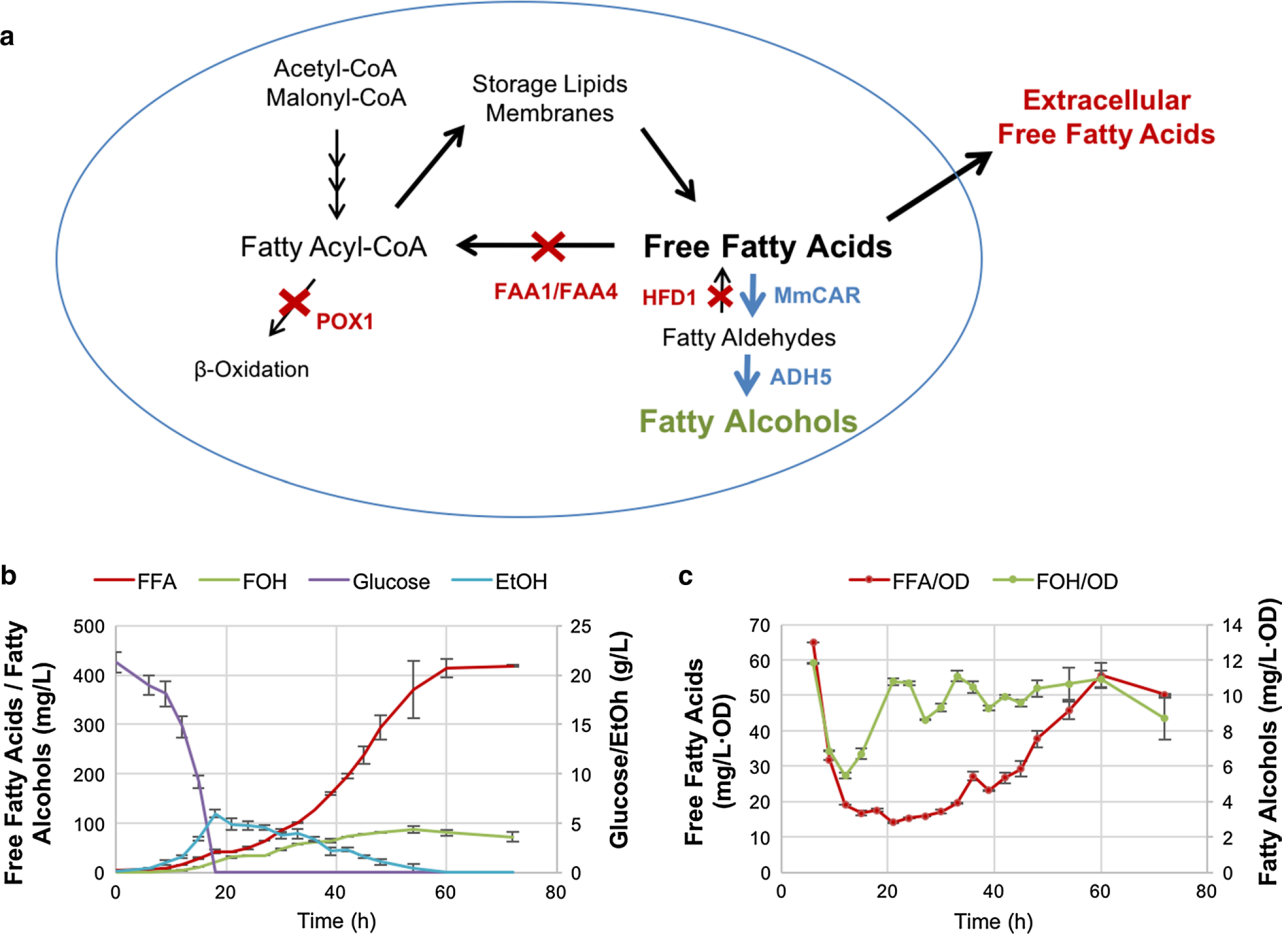
Metabolite profiling of YZFOH1 for production of fatty alcohols from FFAs. **a** Schematic representation of the strain YZFOH1. *POX1*, *HFD1*, *FAA1* and *FAA4* have been deleted in this strain and *MmCAR* and *ADH5* are overexpressed from a 2 µm plasmid (pAOH3). **b** Total FFAs, fatty alcohols (FOH), glucose and ethanol (EtOH) levels in the culture are represented for YZFOH1. Samples were taken every 3–6 h for 72 h from 3 parallel replicate cultures. **c** FFA and fatty alcohol titers divided by the culture OD_600_ at each time point

Before designing and engineering a flux control strategy, the dynamics of metabolite levels throughout the batch culture of the initial fatty alcohol-producing strain was studied. The initial strain YJZ08 (CEN.PK 113-11C *hfd1Δ faa1Δ faa4Δ pox1Δ*) carried deletions in the genes *FAA1* and *FAA4* and as such was unable to reconvert FFAs to acyl-CoAs. Other genes relevant for the process were also deleted such as *POX1*, encoding the first enzyme of the beta-oxidation pathway responsible for the degradation of acyl-CoAs, and *HFD1*, a fatty aldehyde dehydrogenase responsible for conversion of fatty aldehydes into fatty acids. Deletion of *HFD1* has been reported as important for ensuring flux from fatty aldehydes to fatty alcohols in *S. cerevisiae*, since the encoded enzyme efficiently converts produced aldehydes back to their FFA form [12]. YJZ08 was transformed with the 2 µm plasmid pAOH3 for constitutive strong expression of *MmCAR* [11] encoding a carboxylic acid reductase from *Mycobacterium marinum*, which converts long chain FFAs into the respective aldehydes, and *ADH5*, encoding a native alcohol dehydrogenase from *S. cerevisiae*, which efficiently reduces long chain aldehydes to the respective alcohols [5] (Fig. 1a). The resulting strain YZFOH1 was cultivated for 72 h in minimal media with 2% glucose and analyzed by quantification of optical density (OD) and relevant internal and external metabolites, i.e. glucose, ethanol, total FFAs and fatty alcohols. Samples were taken every 3–6 h (Fig. 1b). Due to the ability of fatty acids to form emulsions in the culture media and adsorb to cell membranes, which challenges an accurate distinction between intra- and extracellular FFA, FFA were extracted and quantified from a total volume of culture sample resulting in quantification of total FFA levels.

Measured metabolite profiles showed glucose being entirely consumed at 18 h, while ethanol was gradually formed up to 6 g/L. When glucose was depleted, ethanol started being consumed until its depletion after 60 h. Fatty alcohols and FFAs were constantly produced throughout the culture from either glucose or ethanol as carbon sources. When normalized by the OD values, FFAs showed a constant increase throughout the culture while fatty alcohols reached and oscillated in a plateau from the 21 h time point onwards (Fig. 1c).

The final titers show that from the total formed fatty acids, only 20% were actually converted to fatty alcohols. Furthermore, we observed turbidity in the culture supernatant (not shown) from accumulation of extracellular FFA in form of micelles. This is consistent to what was observed in other studies where similar strains were analyzed [5, 19].

### Designing a dynamic substrate pathway for fatty alcohol production

In order to increase the yield of fatty alcohol production, our primary goal was to fine-tune the FFA to acyl-CoA conversion rates. This would allow FFA levels in the cell to be high enough for optimal conversion to fatty alcohols without reaching the excessive level that causes release of these precursors to the extracellular medium.

Furthermore, strategies explored so far focus on production of fatty alcohols either from acyl-CoA or from FFAs. Here, we explored the possibility of having a combined expression of both pathways that could benefit from high and balanced levels of both fatty acid pools. Production of fatty alcohols from FFAs through MmCAR + Adh5 has a higher potential to achieve high conversion rates compared to conversion from acyl-CoA, since FFAs can accumulate to much higher levels compared to -CoA metabolites [5, 14–16]. On the other hand, the use of acyl-CoA prevents loss and diffusion of fatty acids to the extracellular medium and other subcellular compartments. Acyl-CoA also has the benefit of being the direct product of the fatty acid biosynthesis pathway, while FFAs are a product of lipid recycling with many intermediary steps and higher energy requirements.

We first used the previously described strain YZFOH1 already overexpressing *MmCAR* and *ADH5* and additionally introduced a fatty acyl-CoA reductase gene (*FaCoAR*) from *Marinobacter aquaeolei VT8* [13] expressed from a 2µ plasmid (pAOH9), resulting in strain YZFOH2. This strain would then be able to convert both acyl-CoA directly to fatty alcohols through FaCoAR, convert FFA to fatty aldehydes through MmCAR, and use both Adh5 and FaCoAR to convert fatty aldehydes to fatty alcohols.

While *faa1Δ faa4Δ* strains lack the ability to recycle FFAs back to acyl-CoA, wild type strains expressing both *FAA1* and *FAA4* have a too high acyl-CoA synthetase activity. Therefore, we needed strains that would have different expression patterns of fatty acyl-CoA synthetase so that FFA and acyl-CoA levels could be simultaneously balanced.

Faergeman et al. [20] showed that Faa1 can fully compensate for the loss of Faa4 activity, which means that expression of *FAA1* is sufficient to restore the acyl-CoA activation and import activity lost by the deletion of the two genes.

**Fig. 2:**
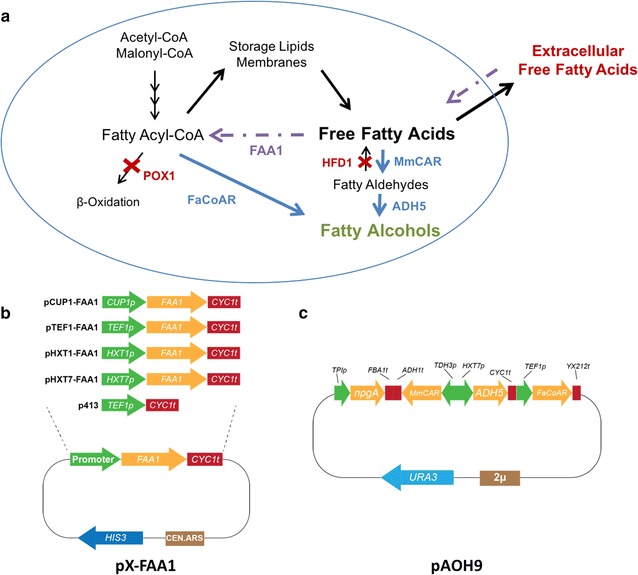
Design of strain producing fatty alcohols from both fatty acyl-CoA and FFAs using dynamically controlled *FAA1* expression. **a** Schematic representation of strain YZFOH2 (YJZ08 pAOH9) expressing *FAA1* under different promoters. *POX1*, *HFD1*, *FAA1* and *FAA4* have been deleted in this strain and *MmCAR*, *ADH5* and *FaCoAR* are overexpressed. **b** Schematic representation of the plasmids constructed for expression of *FAA1*. *FAA1* is expressed from a CEN.ARS plasmid under the control of promoters *CUP1p*, *TEF1p*, *HXT1p* or *HXT7p*, represented as pX-FAA1 for pCUP1-FAA1, pTEF1-FAA1, pHXT1-FAA1 or pHXT7-FAA1, respectively. **c** Schematic representation of the plasmid expressing the enzymes for production of fatty alcohols. npgA, MmCAR, ADH5 and FaCoAR are expressed under strong constitutive promoters from a 2μ plasmid

In our strategy, we wanted to induce *FAA1* expression at specific time points using different native promoters. This would allow us to study the effects of the regulation of FFA and acyl-CoA pools on the conversion of the two precursors to the final product. For that purpose, *FAA1* was cloned on a CEN.ARS plasmid under the control of a copper-induced promoter (*CUP1* promoter) so that expression could be enabled by the addition of Cu^2+^ to the culture medium at specific time points [22, 23]. In parallel, *FAA1* was also put under control of a promoter induced by high glucose and repressed by low glucose concentrations (*HXT1* promoter) and a promoter repressed by high glucose and induced by low glucose conditions (*HXT7* promoter), respectively [24, 25]. As controls, an empty plasmid not expressing *FAA1* (p413) and a plasmid with strong constitutive expression using the *TEF1* promoter (pTEF1-FAA1) (Fig. 2) were used.

### Dynamic control of *FAA1* expression using the copper-inducible promoter *CUP1p*

**Fig. 3:**
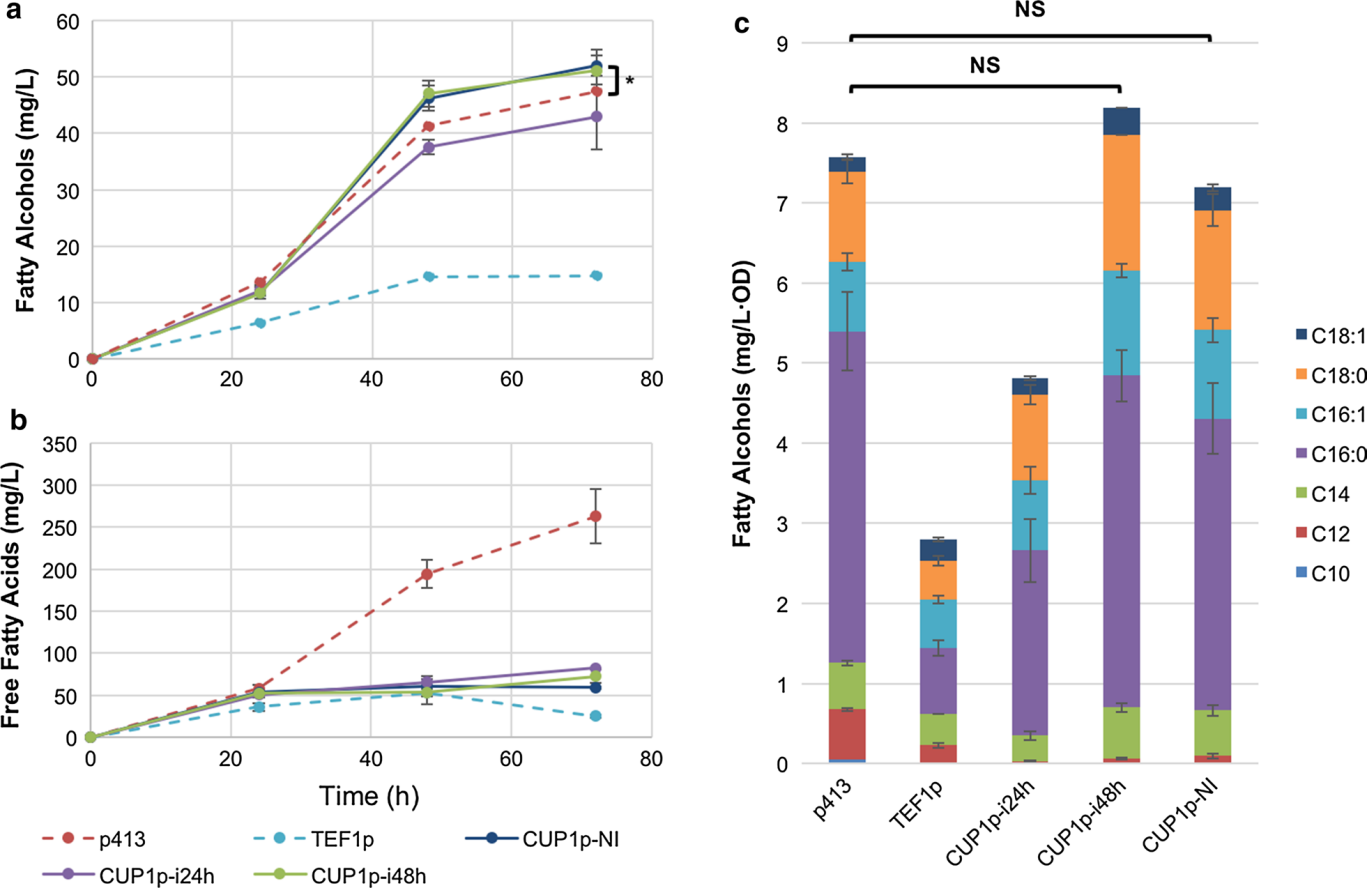
FFA and fatty alcohol production profiles using CUP1p-FAA1. Fatty alcohols (**a**) and FFA (**b**) produced by YZFOH2 expressing *FAA1* under control of the *CUP1* promoter, induced by adding Cu^2+^ at 24 h (CUP1-i24 h) or 48 h (CUP1-i48 h) and without addition of Cu^2+^. For comparison, the same strain without expression of *FAA1* (p413) or with *FAA1* being expressed under control of the *TEF1* promoter were used. **c** Final fatty alcohol specific titers at 72 h normalized to the OD_600_ values. Also shown is the distribution of fatty alcohols in terms of chain length and saturation levels. Experiments were performed with biological triplicates. *p value <0.05, “NS” (not significant): *p* value >0.05 (student’s t test)

YZFOH2 strains expressing *FAA1* under control of the *TEF1* promoter (*TEF1p*-*FAA1*), *CUP1* promoter (*CUP1p*-*FAA1*), or containing an empty plasmid (p413) were cultured for 72 h in minimal media with 2% glucose. Fatty alcohols, FFAs and OD were analyzed at 24, 48 and 72 h (Fig. 3a, b respectively).

Strong constitutive expression of *FAA1* under control of the *TEF1* promoter yielded the lowest amount of FFA and fatty alcohols levels compared with the p413 control. After 72 h of culture, lack of expression of *FAA1* led to production of tenfold more FFAs and around threefold more fatty alcohols compared to constitutive expression of this gene. Also, there was a significant increase in production of both FFAs and fatty alcohol after 24 h in the p413 control strain. The *TEF1p*-*FAA1* strain on the other hand presented a much less steep increase and from the 48 h time point the FFA levels decreased even though fatty alcohol levels did not increase.

Through the use of a copper-inducible promoter *CUP1p*, we induced *FAA1* expression at specific time points by adding 200 µM Cu^2+^ either at 24 or 48 h and compared it with a non-induced culture (no addition of Cu^2+^).

All *CUP1p*-*FAA1* strains produced considerably lower levels of free fatty acids independent of induction time and did not show a decrease of FFA levels after the 48 h time point as it was observed in the *TEF1p*-*FAA1* strain. The non-induced strain produced only 22.6% FFAs compared to the p413 control strain (empty plasmid). When induced at 24 and at 48 h, FFA production levels are 27.7 and 31.1%, respectively, compared with the p413 control strain.

Inducing expression of *FAA1* at 48 h resulted in the highest fatty alcohol titer of 52 mg/L, which is approximately the same titer achieved by the non-induced strain, and higher than the control strain of 47.4 mg/L. Inducing expression of *FAA1* at 48 h resulted in the highest specific fatty alcohol titers of 8.2 mg/L·OD, and while this is slightly higher than the control strain of 7.6 mg/L·OD, the difference between the two is not statistically significant (*p* > 0.05) (Fig. 3c). Furthermore, no turbidity resulting from FFA micelles was observed in the culture supernatant for *TEF1p*-*FAA1* or any *CUP1p*-*FAA1* strains, while presence of this was evident for the p413 control strain, indicating a very significant reduction in excreted FFAs.

These results suggest that presence of low Faa1 levels might be beneficial through most of the culture for balancing the fatty acid pools. However, it is clear that a strong induction of expression early in the process causes a too high flux towards conversion of FFA to acyl-CoA, therefore compromising pathway balance.

### Dynamic regulation of FFA and acyl-CoA pools using glucose-regulated promoters

As an alternative strategy to copper-induced promoters, we sought to control *FAA1* expression with glucose-responsive promoters, which allows syncing expression levels with growth phases and carbon source usage. For this purpose, we chose the hexose transporter gene promoters *HXT1p* and *HXT7p*. The promoter *HXT1p* is induced at high glucose concentration and repressed at low glucose concentration [24]. This means that in the *HTX1p*-*FAA1* system Faa1 is produced during the initial high glucose phase of the batch culture (approximately the first 18 h, Fig. 1b), followed by a tight repression during the ethanol phase, which leads to very low levels of Faa1 present in the cytosol through most of the culture. On the other hand, *HXT7p* is induced under low glucose conditions, while being repressed at high glucose levels [25]. This way, *FAA1* is only expressed in the end of the glucose phase when glucose levels are very low so that FFAs are converted to acyl-CoA mostly during the ethanol phase.

**Fig. 4:**
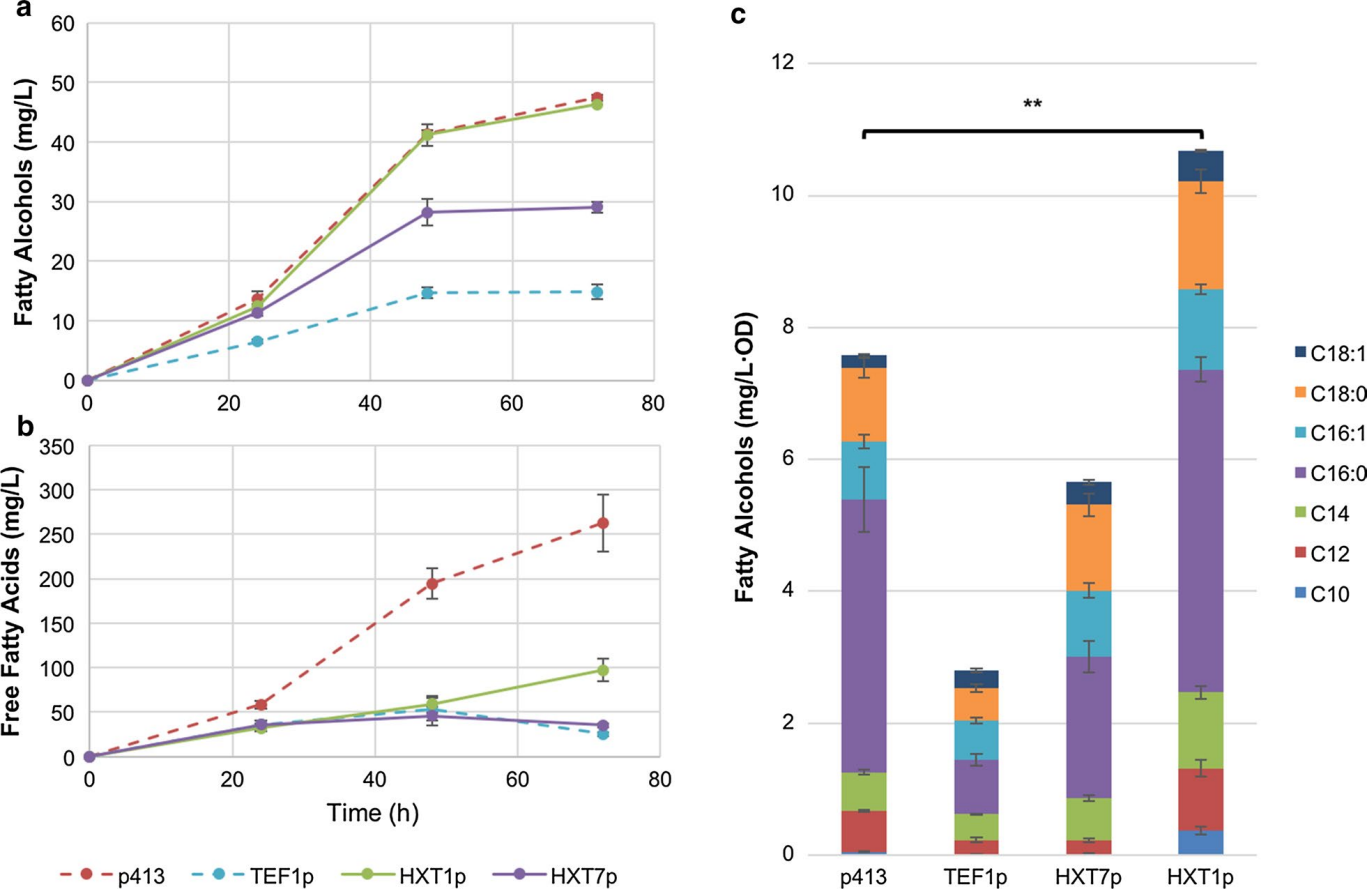
FFA and fatty alcohol production profiles using *FAA1* expressed under control of promoters *HXT1p* or *HXT7p*. Fatty alcohol (**a**) and FFA titers (**b**) produced by YZFOH2 expressing *FAA1* under control of the *HXT1* or *HXT7* promoters. For comparison, the same strain without expression of *FAA1* (p413) or with *FAA1* being expressed under control of the *TEF1* promoter were used. **c** Final fatty alcohol titers at 72 h normalized to the total OD_600_ values. Also shown is the distribution of fatty alcohols in terms of chain length and saturation levels. **p value <0.005 (student’s t test)

OD, fatty alcohol and FFA levels were analyzed at 24, 48 and 72 h for these strains (Fig. 4a, b respectively). Final FFA levels at 72 h showed a decrease by 63 and 87% for *HXT1p*-*FAA1* and *HXT7p*-*FAA1* strains, respectively. *HXT7p*-*FAA1* showed a small decrease in FFA levels after 48 h, which is similar to *TEF1p*-*FAA1,* while in the *HXT1p*-*FAA1* system, FFAs showed a linear increase throughout the culture. Fatty alcohol final titers in the *HXT7p*-*FAA1* strain were only 61% of the p413 control strain while the *HXT1p*-*FAA1* strain achieved a fatty alcohol titer of 43.6 mg/L, the same level as the p413 control strain. Furthermore, the specific fatty alcohol titer (production level per OD) of *HXT1p*-*FAA1* was improved by 41% compared with the control strain (Fig. 4c), which suggests a higher cellular metabolic flux toward fatty alcohol biosynthesis.

Analysis of chain length and saturation level of formed fatty alcohols shows that increased expression levels of *FAA1* decreased the percentage of C16:0 fatty alcohols produced and increased the percentage of unsaturated fatty alcohols C16:1 and C18:1. In addition, there was a 4.5-fold increase in decanol production exclusively in the *HXT1*-*FAA1* strain (Table 3).

**Table 3:** Fatty alcohol distribution in strains expressing FAA1 under control of different promoters

% Total fatty alcohols							
Strain^a^	C10	C12	C14	C16:0	C16:1	C18:0	C18:1
p413	0.65 ± 0.11	8.25 ± 0.24	7.67 ± 0.47	54.62 ± 6.48	11.52 ± 1.43	14.74 ± 1.91	2.55 ± 0.33
*HXT1p*-*FAA1*	3.50 ± 0.52	8.81 ± 1.20	10.81 ± 0.89	45.81 ± 1.73	11.37 ± 0.66	15.29 ± 1.70	4.40 ± 0.11
*HXT7p*-*FAA1*	0.38 ± 0.04	3.62 ± 0.52	11.25 ± 0.76	38.08 ± 4.21	17.64 ± 2.05	22.98 ± 2.98	6.06 ± 0.77
*TEF1p*-*FAA1*	0.64 ± 0.10	7.48 ± 1.23	14.05 ± 0.14	29.49 ± 3.35	21.48 ± 1.59	17.33 ± 2.24	9.54 ± 0.94

^a^Experiment is a result of biological triplicates, standard deviation is presented for each value

Since we observed a substantial decrease in measured FFAs while fatty alcohol levels were kept stable, we investigated the possibility of fatty acids being accumulated in other forms such as storage lipids or phospholipids. For that, we analyzed the total intracellular lipid content on these strains. Between the control strain p413 and *TEF1p*-*FAA1*, the lipid profile was radically changed. It is clear that expression of *FAA1* led to a higher accumulation of storage and membrane lipids, since *TEF1p*-*FAA1* showed a 45% increase in sterol esters, an 86% increase in triacylglycerols and at least a threefold increase in every form of phospholipid measured. The *HXT7p*-*FAA1* system showed a lipid profile very close to *TEF1p*-*FAA1*, while the profile of *HXT1p*-*FAA1* was very close to the control strain p413, with the exception of sterol esters, which in *HXT1p*-*FAA1* was increased by 37% (Additional file 1: Figure S2).

Growth kinetics for the strains p413, *TEF1p*-*FAA, HXT1p*-*FAA1* and *CUP1p*-*FAA1* (not induced by CuSO_4_) were also studied by measuring OD_600_ values at several timepoints over 72 h. Growth rates on glucose ranged from 0.16 h^−1^ for *HXT1p*-*FAA1* to 0.19 h^−1^ for *TEF1p*-*FAA1* but with no statistically significant difference (p value >0.05 for t test analysis) between p413 control and any other strain. Final OD_600_ values at 72 h for *HXT1p*-*FAA1* were of 4.9, which is lower than the values reached by the other strains (6.3 for p413 strain, 5.7 for *TEF1p*-*FAA1*, *7.3 for CUP1p*-*FAA1)* (Additional file 1: Figure S3).

## Discussion

Metabolic balancing is essential for production of target molecules with high yield. In this study, metabolic profiling of a fatty alcohol producing strain YZFOH1 (Fig. 1) suggested a limitation in fatty alcohol production and accumulation of fatty acids during the course of the batch culture. The limitation in conversion of FFAs to fatty alcohols results in secretion of FFA to the culture medium, causing loss of precursors needed for fatty alcohol biosynthesis and consequential loss of yield.

We thus engineered fatty alcohol-producing strains using dynamically controlled expression of the fatty acyl-CoA synthetase gene *FAA1* in order to balance the levels of FFAs and acyl-CoA in the cell. This allowed us not only to optimize the intracellular fatty acid levels for conversion to the desired product, but also to explore a potential additive or synergistic effect of using both acyl-CoA and FFA-consuming pathways for fatty alcohol production.

Controlling expression of *FAA1* using the *CUP1* promoter with different induction times resulted in FFA production levels approximately between 20 and 30% of the levels measured for the control strain. On the cases of late induction at 48 h and using only basal expression levels the final fatty alcohol titers were increased by approximately 10% (Fig. 3). On the other hand, early expression of *FAA1* was detrimental to the final fatty alcohol titers and yield. A similar effect could be observed by expressing *FAA1* under control of a *HXT1* promoter, where FFA levels were drastically reduced while fatty alcohol levels per biomass were improved. The *CUP1* promoter has been previously characterized and in a single-copy plasmid it is known to have basal expression levels up to 7% (based on enzyme activity level) compared to induction with 100 µM Cu^2+^ [22, 23]. Furthermore, the minimal culture medium used in this study contains approximately 6 µM Cu^2+^, therefore contributing to a moderate expression level of *FAA1* on the non-induced culture. These results suggest that constitutive basal expression levels of the *CUP1* promoter in our culture conditions produced enough enzyme to regulate the acyl-CoA/FFA levels in a favourable way. The later (48 h) induced expression or not-induced expression of *FAA1* resulted in much higher fatty alcohol production compared to the earlier induction (24 h) (Fig. 3c), which suggested that sustained high expression of *FAA1* created a too high flux of FFA conversion to acyl-CoA, reducing FFA supply for fatty alcohol biosynthesis. This was corroborated when *FAA1* was expressed under control of the *HXT7* promoter. Although main expression of *HXT7* promoter occurs only at the end of the glucose phase, basal expression levels are quite high when compared with *HXT1p* [24, 25], which creates a phenotype much closer to *TEF1p*-*FAA1*.

Overall interpretation of the results indicates that *TEF1p*-*FAA1*, *HXT7p*-*FAA1* and early induced *CUP1p*-*FAA1* express high levels of *FAA1*. High levels of FAA1 expression created a high flux of FFA towards acyl-CoA formation, which lowered FFA availability for fatty alcohol production through *MmCAR*. Although this could promote higher flux through FaCoAR due to a higher substrate supply, it is generally accepted that acyl-CoA cannot be accumulated in high concentrations due to limited -CoA availability in the cell. Additionally, an increase of acyl-CoA levels in yeast has been previously shown to be related with feedback inhibition of fatty acid biosynthesis at the level of the acyl-CoA carboxylase Acc1 [26], and therefore having a negative effect on fatty acid and fatty acid-derived chemicals production. Furthermore, in contrast to FFAs, acyl-CoA is consumed in many competing reactions, which can be demonstrated by the increased levels of certain storage and phospholipids in those strains with higher *FAA1* expression.

The *HXT1* promoter, on the other hand, allows for expression of *FAA1* in high glucose conditions, which is the case during the very early stage of the batch culture, not longer than 15 h of culture. From that point on, *HXT1p* presumably keeps the expression level of *FAA1* low, which apparently represents a favourable flux balance to maintain the acyl-CoA/FFA pools for efficient conversion of these metabolites into fatty alcohols.

When the distribution of produced fatty alcohols was analyzed in the resulting strains, it was possible to observe a shift in saturation levels and chain length related to the promoter strength expressing FAA1. By respective order, *HXT1p*-*FAA1*, *HXT7p*-*FAA1* and *TEF1p*-*FAA1* strains showed a higher ratio of C16:1/C16:0 and C18:1/C18:0 as well as a higher percentage of C14 fatty alcohols compared with the control strain p413 (Table 3). This is probably a result of Faa1 specificity, since most enzymes have higher affinity towards specific fatty acid chain lengths and saturation levels. Affinity of Faa1 towards different fatty acids would change the balance of corresponding acyl-CoA/FFA for those fatty acids in question and therefore influence the final product distribution. This is a valuable tool if production of a specific fatty alcohol is desired.

Furthermore, variations on *FAA1* expression levels seem to have a minimal impact on growth kinetics. A small decrease in OD_600_ at 72 h was observed for *HXT1*-*FAA1*, but not for *CUP1*-*FAA1.* Since *HXT1*-*FAA1* shows a higher level of accumulated FFA at 72 h compared to *CUP1*-*FAA1* and nearly identical fatty alcohol titers, the growth defect might suggest a certain toxicity from high FFA levels during stationary phase when *FAA1* is present at a minimal level.

Together, our set of experiments indicates a need for tight regulation of *FAA1* expression in order to achieve an optimal balance of acyl-CoA and FFAs for fatty acid production from a combined use of our two pathways. We see a favourable effect in using very low levels of *FAA1* expression, which can be hard to achieve using native promoters whose activity may be influenced by many factors. Here, the use of technologies that allow for a more predictable and fine-tuned expression such as synthetic promoters [27, 28], CRISPRi [29, 30] and acyl-CoA biosensors [31] for dynamic gene expression could be beneficial for future optimisation on similar systems.

Nevertheless, our final strain represents an improvement over the original one due to the capacity of being able to produce fatty alcohols in similar or slightly improved final titers but with better product-to-biomass yield and without high production of FFA by-products. Further improvement of these strains for increased performance is now possible since precursor accumulation is not an issue that compromises yield.

## Conclusions

In this work, we exemplified how one can address the arising problem of unbalanced fluxes in a synthetic pathway. We addressed a situation where kinetics of downstream steps is a limiting factor on the product formation, which leads to accumulation and loss of precursors to the extracellular medium, compromising final yields where these are a fundamental aspect of the process. In many cases accumulated precursors might end up as by-products that comprise the entire production process or represent a major carbon loss. In this aspect, pathway fine-tuning is a fundamental asset for applicability of existing pathway prototypes in microbial cell factories that must find its place in the design process.

On the other hand, the work also provides general knowledge for the development of cell factories for fatty acid-derived products. Fatty acid recycling and regulation of FFA/acyl-CoA interconversion is a central aspect, which is not completely understood and our analysis provide new insight into how fluxes are controlled in this pathway.

## Additional file

**Additional file 1.**  Additional figures and tables.
