# Supplementary material for: Dynamic regulation of fatty acid pools for improved production of fatty alcohols in Saccharomyces cerevisiae
Source: Microb Cell Fact. 2017 Mar 15;16:45. doi: 10.1186/s12934-017-0663-3 (PMC5353878; doi:10.1186/s12934-017-0663-3)
Supplement: Supplementary file 1 — Additional file 1. Additional figures and tables. [file 12934_2017_663_MOESM1_ESM.pdf]

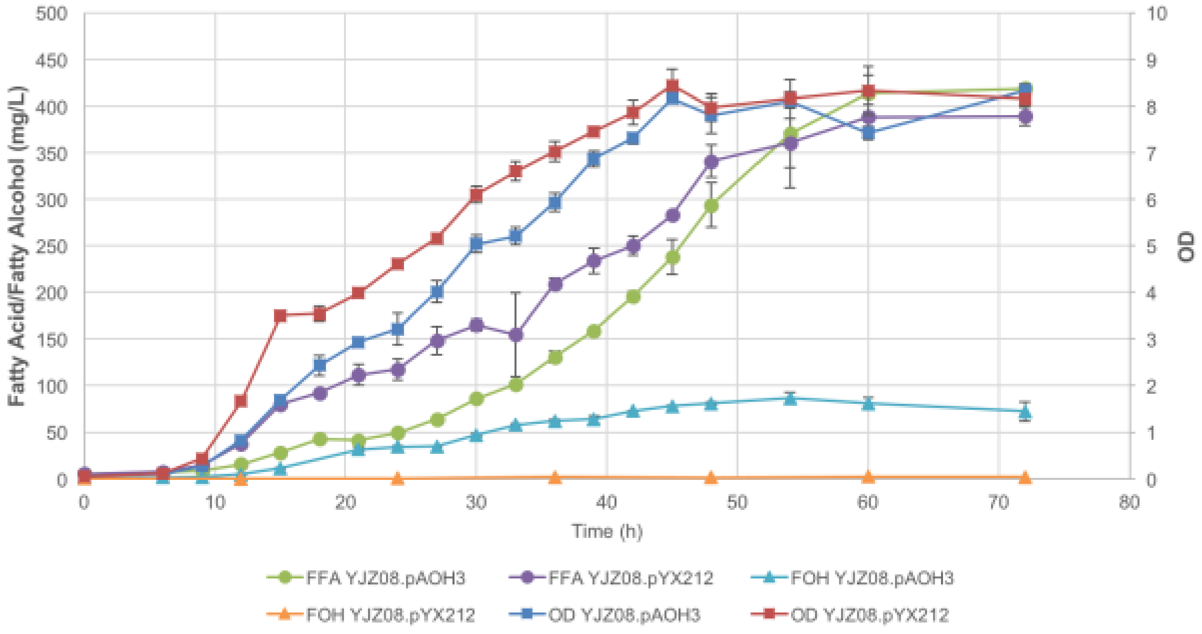

Supplementary Figure S1. Growth, Free Fatty Acid and Fatty Alcohol production profile of YJZ08-pAOH3 (YZFOH1) vs YJZ08-pYX212. Total FFAs, fatty alcohols (FOH), glucose and OD in the culture are represented for YJZ08-pAOH3 (expressing *MmCAR* and *ADH5* for fatty alcohol production) and YJZ08-pYX212 (empty plasmid control). Samples were taken every 3 to 6 hours for 72 h from 3 parallel replicate cultures.

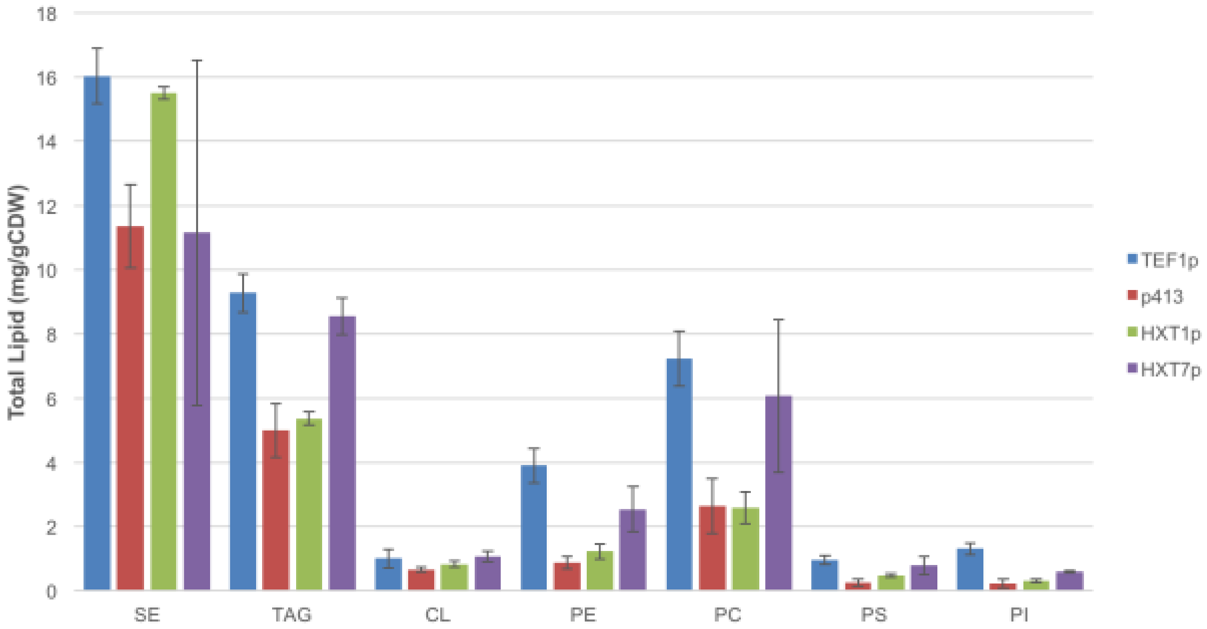

Supplementary Figure S2. Lipid profile of YZFOH2 expressing *FAA1* under control of different promoters. Sampling of YZFOH2 culture in minimal medium was performed at 72 h. 10 mg of dry biomass were extracted by the chloroform:methanol method and analyzed by HPLC-CAD. SE: sterol esters; TAG: triacylglycerols; CL: cardiolipin; PE: phosphatidylethanolamine; PC: phosphatidylcholine; PS: phosphatidylserine; PI: phosphatidylinositol.

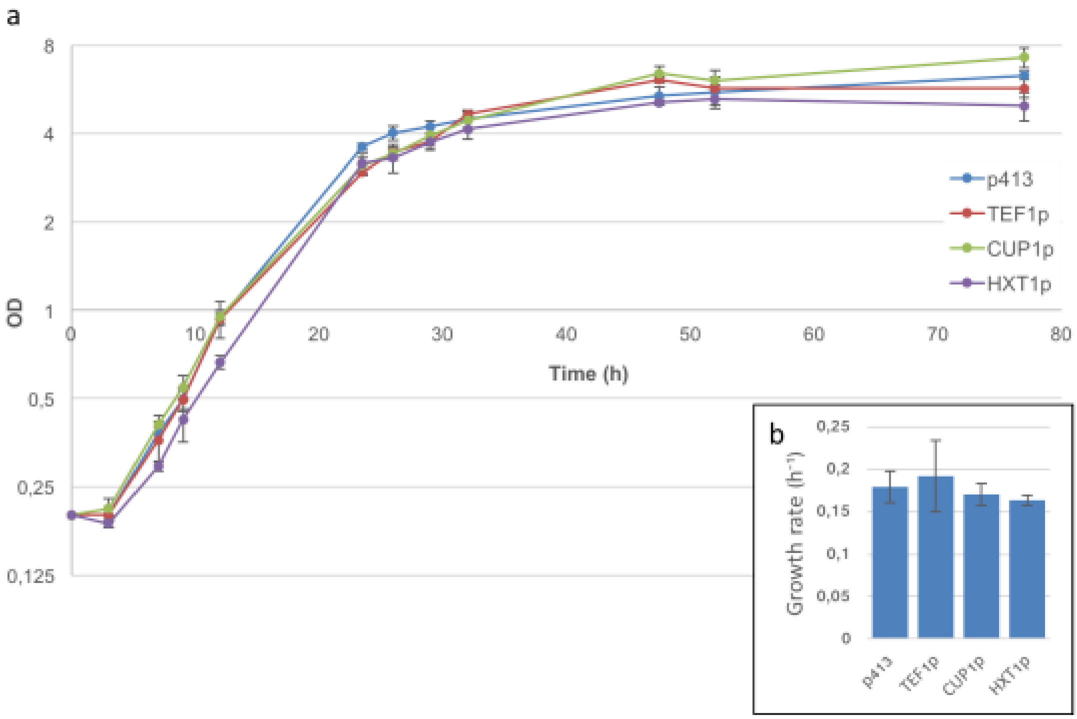

Supplementary Figure S3. Growth kinetics of YZFOH2 expressing *FAA1* under control of different promoters. a) Optical density at 600nm (OD) was measured at specified time points for 3 different cultures for each strain (*FAA1* expressed under control of *TEF1p*, *HXT1p* or *HXT7p* promoters or using the p413 empty plasmid not expressing *FAA1*) cultivated in shake flasks. OD axis is shown in a logarithmic scale b) Growth rate during growth on glucose for the strains specified before. Growth rate was calculated using the time points between 6 h and 12 h of the different cultivations.

Supplementary Table S1. DNA oligos used for amplification and cloning.

Primer Name	Sequence
p416-RV	GAGCTCCAGCTTTTGTTC
p416-FW	GTCATGTAATTAGTTATGTCACG
CTR3prom-F	cctcactaaagggaacaaaagctggagctcTTCAACAGAAATTGAGAAACAAATTTAC
CTR3prom-R	atattgagcaaccatCTTTGTATAGCCCTTAAATGTGTTTTC
CTR3-FAA1-F	aagggctatacaaagATGGTTGCTCAATATACCG
CUP1prom-F	cctcactaaagggaacaaaagctggagctcTTTAAAACACTTTTGTATTATTTTTC
CUP1prom-R	atattgagcaaccatTTTATGTGATGATTGATTGATTG
CUP1-FAA1-F	caatcatcacataaaATGGTTGCTCAATATACCG
HXT1prom-F	cctcactaaagggaacaaaagctggagctcACTATTATTCCTCCGAGAAAAC
HXT1prom-R	atattgagcaaccatGATTTTACGTATATCAACTAGTTGAC
HXT1-FAA1-F	gatatacgtaaaatcATGGTTGCTCAATATACCG
HXT7prom-F	cctcactaaagggaacaaaagctggagctcAATAGTACTCTCATCGCTAAGATC
HXT7prom-R	atattgagcaaccatTTTTTGATTAAAATTAAAAAAACTTTTTG
HXT7-FAA1-F	aattttaatcaaaaaATGGTTGCTCAATATACCG
TEF-FAA1-F	aattacaaatctagaactagtggatcccccATGGTTGCTCAATATACCG
ADH-FAA1-F	gctggccgctctagaactagtggatcccccATGGTTGCTCAATATACCG
FAA1-R	atgtaagcgtgacataactaattacatgacTTAAGACGAACTATAAACGGC
FAA1-SmaI-R	gataagcttgatatcgaattcctgcagcccTTAAGACGAACTATAAACGGC
